# Cellular and extracellular vaginal changes following murine ovarian removal

**DOI:** 10.14814/phy2.15762

**Published:** 2023-08-07

**Authors:** Jennifer M. McCracken, Gisele A. Calderon, Quynh N. Le, Natasha M. Faruqui, Swathi Balaji, Julie C. E. Hakim

**Affiliations:** ^1^ Department of Obstetrics and Gynecology Division of Pediatric and Adolescent Gynecology Texas Children's Hospital, Baylor College of Medicine Houston Texas USA; ^2^ Department of Surgery Division of Pediatric Surgery Texas Children's Hospital, Baylor College of Medicine Houston Texas USA

**Keywords:** collagen, estrogen signaling, hyaluronan, inflammation, vagina

## Abstract

Loss of estrogen as a result of aging, pelvic cancer therapy, genetics, or eating disorders affects numerous body systems including the reproductive tract. Specifically, a chronic hypoestrogenic state fosters debilitating vaginal symptoms like atrophy, dryness, and dyspareunia. Current treatment options, including vaginal estrogen and hyaluronan (HA), anecdotally improve symptoms, but rectifying mechanisms are largely understudied. In order to study the hypoestrogenic vaginal environment, in particular the extracellular matrix (ECM), as well as understand the mechanisms behind current treatments and develop new therapies, we characterized a reliable and reproducible animal model. Bilateral ovariectomies (OVX) were performed on 9‐week‐old CD1 mice. After 1 month of estrogen loss due to ovarian removal, a phenotype that is similar to human vaginal tissue in an estrogen reduced state was noted in mice compared to sham‐operated controls. The uterine to body weight ratio decreased by 80% and vaginal epithelium was significantly thinner in OVX compared to sham mice. Estrogen signaling was altered in OVX, but submucosal ERα localization did not reach statistical differences. HA localization in the submucosal area was altered and CD44 expression decreased in OVX mice. Collagen turn‐over was altered following OVX. The inflammation profile was also disrupted, and submucosal vaginal CD45^+^ and F4/80^+^ cell populations were significantly reduced in the OVX mice. These results show altered cellular and molecular changes due to reduced estrogen levels. Developing new treatments for hypoestrogenic vaginal symptoms rely on better understanding of not only the cellular changes, but also the altered vaginal ECM environment. Further studies using this mouse model has the potential to advance women's vaginal health treatments and aid in understanding the interplay between organ systems in both healthy, aged, and diseased states.

## Graphical abstract

Murine ovariectomy resulted in decreased circulating estrogen as evidence by smaller uterine: BW ratio. The hypoestrogenic vaginal state displayed altered estrogen signaling, hyaluron localization, collagen turn over, and inflammatory profile.

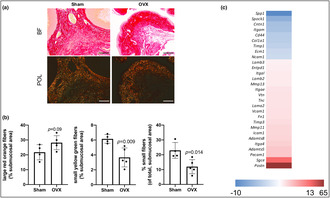

## 1 INTRODUCTION

Each year, approximately 1.3 million women in the United States will reach menopause at an average of 51 years of age (the onset of the menopausal state can be endured for years) (Peacock & Ketvertis, 2022). Menopause affects multiple organ systems and leads to symptoms such as hot flashes, sleep disruptions, and mood changes, as well as increased risk of heart disease, and altered vaginal environment (Maslov et al., 2019; Peacock & Ketvertis, 2022). Changes in the epithelium and microbiota of the vagina can present clinically as vulvovaginal atrophy, vaginal dryness, and dyspareunia, which may exacerbate a woman's menopausal transition (Muhleisen & Herbst‐Kralovetz, 2016). One of the most common and debilitating consequences of menopause and aging is the loss of estrogen. Low estrogen due to conditions both related and unrelated to menopause like eating disorders (Schorr & Miller, 2017), cancer treatments impacting ovaries (Guidozzi, 2013), or certain genetic disorders (Nippita & Baber, 2007) may result in similar symptoms, including loss of vaginal lubricity and susceptibility to gynecologic functions. Managing vaginal atrophy symptoms can negatively impact women's mental and physical health, personal relationships, and productivity (Assaf et al., 2017; Parish et al., 2013). Beyond its cost on quality of life, these symptoms also present a great economic burden to the healthcare system through numerous physician visits, medications, laboratory evaluations, and management of treatment side effects (Assaf et al., 2017). Despite the personal and economic burden of vulvovaginal atrophy, there is minimal understanding of the basic biology of the hypoestrogenic vaginal tissue microenvironment.

The integrity of vaginal tissue is heavily influenced by the presence of hormones and their signaling. Vulvovaginal tissue presents as an estrogen‐rich environment with cells expressing both estrogen receptor (ER) α and ERβ (Chen et al., 1999; Dupont et al., 2000). These ERs, when activated, play a role in forming the vaginal rugae by promoting the proliferation of epithelium, smooth muscle fibers, and collagen (Palacios, 2009). The vaginal rugae increase the surface area of the vagina allowing for more stretch and extension during sexual stimulation and parturition (Ashton‐Miller & Delancey, 2009; Gandhi et al., 2016). Hypoestrogenism causes the vaginal epithelium to become thinner with fewer folds, dryer, and less elastic: factors that make vulvovaginal tissues of women more prone to injury and inflammation after estrogen loss (Naumova & Castelo‐Branco, 2018).

Current treatment options include different forms of estrogen supplementation (Muhleisen & Herbst‐Kralovetz, 2016). Low‐dose estrogen is suggested to help restore the flattened, estrogen‐deprived vaginal and urethral epithelia that leave menopausal women more susceptible to infection (Bachmann & Nevadunsky, 2000; North American Menopause S, 2007). A study reported that 74% of women with vulvovaginal atrophy experienced relief by the end of 1 year of oral estrogen replacement therapy (Gass et al., 2011). In a meta‐analysis study, local estrogen treatment was found to be more effective by eliminating 80%–90% of vaginal atrophy cases compared to 75% when using systemic hormone replacement therapy (HRT) (Cardozo et al., 1998). Previous studies also showed accelerated and complete regenerative vaginal surgical wound healing mediated by estrogen signaling (McCracken et al., 2021). Between systemic and vaginal estrogen therapy, the latter has been the recommended form of treatment for those with only genitourinary menopausal symptoms, as it administers a lower and localized dose of estrogen helping to prevent the risk of hyperplasia or carcinoma (Gandhi et al., 2016). However, since there is a limited understanding of the changes that occur in the cellular, molecular and ECM milieu of the hypoestrogenic vagina, we do not completely understand what aspects of the pathology estrogen supplementation treatments address.

Healthy vaginal tissue contains ubiquitous hyaluronan (HA), a glycosaminoglycan within the extracellular space of the submucosal area which aids in tissue hydration and lubrication. Therefore, vaginal HA has become an alternative, nonhormonal treatment for genitourinary menopausal symptoms. It alleviates vaginal dryness and dyspareunia by retaining water molecules, increasing tissue hydration, and inducing physiologic changes which reverse many vaginal issues related to loss of estrogen (Kagan et al., 2019; Salwowska et al., 2016). Studies comparing vaginal HA to estradiol treatment showed that both treatments significantly improved epithelial atrophy, decreased vaginal pH, and induced maturation of vaginal epithelium (Dos Santos et al., 2021; Ekin et al., 2011). The vaginal HA treatment significantly improved symptoms of atrophic vaginitis and showed that HA may be a viable option when estrogen therapy is contraindicated (Ekin et al., 2011). While the results present HA as a promising alternative to HRT, each of the five primary studies measured data in different ways, and additional research studies are needed to assess the efficacy and longevity of this treatment (Dos Santos et al., 2021).

Given the prevalence of genitourinary menopausal symptoms and their impact on quality of life, a thorough model of the basic biology of these vaginal symptoms is necessary to serve as a baseline to test effective and long‐lasting treatments. Removing both ovaries from a mouse has been shown to effectively lower circulating hormones, primarily estrogen, and cause vaginal atrophy particularly as it relates to vaginal epithelium (Ceccarelli et al., 2014). Taken together, the aim of this study was to utilize this murine model of reduced estrogen state to understand the relationship of estrogen, inflammation, and vaginal extracellular matrix (ECM). The ECM and innate immune perturbations were evaluated in this murine model by characterizing vaginal tissue remodeling post ovariectomy (OVX). Similar to other reports, epithelial changes occurred after ovarian removal along with vaginal atrophy that resulted in dysregulated and altered tissue architecture and inflammatory cells. Increasing the understanding of the altered cellular and tissue environment in a hypoestrogenic model will enhance the development of therapeutic targets caused by low estrogen levels.

## 2 MATERIALS AND METHODS

### 2.1 Animal IACUC and Ethics Statement

Female CD1 mice, 9 weeks of age, were purchased from Baylor College of Medicine (BCM) Center for Comparative Medicine. They were socially housed, exposed to 12 h light/12 h dark cycles, and given free access to standard food and water. All procedures described herein were approved by BCM Institution for Animal Care and Use Committee, AN‐7475, and completed in compliance with all recommendations in the Guide for the Care and Use of Labratory Animals of the National Institute of Health.

### 2.2 Animal model and tissue harvest

Mice were randomly separated into two groups (*n* = 4–5 per group): sham and OVX. Mice were anesthetized using 0.5 L/min isoflurane. Bilateral, 0.5 cm incisions were created in the dorsal flank. In Sham control mice, the ovaries were visualized, then the incisions were closed with 5‐0 vicryl and/or Dermabond skin glue. In OVX mice, once the ovaries were visualized, the ovarian ligament was clamped and cauterized, and each ovary was excised. The incisions were again closed with 5‐0 vicryl and/or Dermabond skin glue. Sham and OVX mice received standard postoperative care including observation and administration of analgesics for 3 days following the procedure.

Four weeks after the surgical procedures, all mice were weighed and euthanized, and the reproductive tract was harvested. The uterine horns were isolated and weighed to calculate the uterine horns to body weight ratio, a surrogate marker of estrogen circulation (Elliot et al., 2003). The vaginal canal of each mouse was harvested from cervix to introitus. Tissue was divided along the longitudinal axis with a portion fixed in 10% formalin and the remainder snap frozen in liquid nitrogen for future analysis.

### 2.3 Histology

Tissue fixed in 10% neutral buffered formalin was dehydrated through a series of graded ethanol and xylenes and embedded in paraffin (FFPE). Sections were cut to 5 μm, rehydrated, and stained using standard hematoxylin and eosin.

To determine epithelial thickness, 20X images were taken using Leica DMi8 microscope with a Leica DFC4500 camera and Leica Application Suite X (LAS‐X). ImageJ software was used to measure the epithelial thickness along 10 points of each image, 3 images per mouse were analyzed and all measurements were averaged.

HA staining was evaluated using HA binding protein (HABP) and a protocol adapted from Cleveland Clinic's Programs of Excellence in Glycosciences. Briefly, FFPE tissue sections were rehydrated in a series of graded ethanol. Slides were equilibrized in PBS, endogenous avidin and biotin were blocked (SP‐2001, Vector Labs). Slides were incubated in normal goat serum (150 μL/10 mL) followed by biotinylated HABP (385911, 1:500, Calbiochem) for 1 h. After washing, the signal was amplified using avidin/biotin complex (PK‐6101, Vector Labs) and detected using DAB (SK‐4105, Vector Labs). Slides were counterstained using hematoxylin (K8008, Agilent). 20X (*n* = 3 per mouse) images were taken using Leica DMi8 microscope with a Leica DFC4500 camera and LAS‐X. ImageJ was used to determine the percent positive area above a set threshold which was kept constant for each image and reported as percent positive area (positive area/total submucosal area × 100%).

Collagen expression was evaluated using Picrosirius red (PSR) staining. Slides were stained in PSR solution (0.1 gm Sirius Red F3B, C.I. 35782, in 100 mL saturated aqueous solution of picric acid) for 1 h following deparaffinization to water. After two rinses with acidified water (5 mL glacial acetic acid in 1‐L deionized water), samples were dehydrated with three changes of 100% ethanol. Finally, slides were cleared with xylene and cover slipped for subsequent imaging. 20X (*n* = 3 per mouse) images, under both bright field and circular polarized light, were taken using Leica DMi8 microscope with a Leica DFC4500 camera and LAS‐X. ImageJ was used to determine total submucosal area. Using the polarized light images, color masks were created to measure positive red area and positive yellow/green area as previously described (Zerbinati & Calligaro, 2018) with the following parameters, red: hue 0–34, saturation 0–255, brightness 75–255, yellow/green: hue 42–118, saturation 0–255, brightness 43–255. Data were reported as percent positive area for large, Type I collagen (red) or small, Type III collagen (yellow/green) fibers. The percent of small fibers was calculated using the following formula:

$$\frac{\left(\text{Area of yellow fibers}\right)}{\left(\text{Area of yellow fibers}+\text{Area of}\;\mathrm{red}\;\text{fibers}\right)}\times 100$$

Immunohistochemistry (IHC) was used to localize ERα, HA receptor (CD44), and inflammation markers (CD45, F4/80) as described previously (McCracken et al., 2021). Briefly, FFPE sections were rehydrated in xylenes and graded ethanol, and antigen retrieval was done using low target retrieval reagent (K8005, Agilent) and the PT Link retrieval system (Agilent). IHC was completed using Dako Autostainer Link 48 (Agilent) and appropriate reagents including peroxidase and alkaline phosphatase blocking reagent (S2003, Agilent), antibody diluent (S0809, Agilent), anti‐rabbit (K4009, Agilent), 3,3′‐diaminobenzidine to visualize (DAB, K3468, Agilent), and hematoxylin as counterstain (K8008, Agilent). Primary antibodies used were CD44 (ab157107, 1:7500, Ab‐cam, Cambridge, MA), CD45 (ab10558, 1:2000, AbCam), ERα (ab32063, 1:500, Ab‐cam), and F4/80 (MF480000, 1:5000, ThermoFisher). 20X (*n* = 3 per mouse) images were taken using Leica DMi8 microscope with a Leica DFC4500 camera and LAS‐X. ImageJ was used to determine submucosal area and positive cell number to calculate number of cells per submucosal area.

### 2.4 Gene expression

RNA was isolated from flash frozen vaginal tissue using PureLink RNA Mini kit (12183025, ThermoFisher) according to the manufacturer's instructions, including DNA elimination step.

For gene array data sets, RNA was pooled from each group for a total of 300 ng of RNA and was reverse transcribed to cDNA using RT^2^ (Maslov et al., 2019) First Strand Kit according to the manufacturer's instructions (330404, Qiagen). The following gene arrays were completed: mouse ER signaling (PAMM‐005Z, Qiagen), mouse ECM and adhesion molecules (PAMM‐013Z, Qiagen), and mouse inflammatory cytokines and receptor (PAMM‐011Z, Qiagen). Analyses were completed using the Qiagen GeneGlobe Analysis platform and expressed as fold over sham mice.

For standard qPCR, 330 ng of RNA was reverse transcribed using High‐Capacity RNA‐to‐cDNA kit (43–874‐06, Fisher Scientific). Samples were performed in duplicate using Power SYBR Green PCR Master Mix (43–676‐59, Fisher Scientific) and Bio‐Rad CFX384 thermocycler. Primer sequences used are listed in Table 1. Data were analyzed using the ΔΔ*C*_*t*_ method and expressed as fold change over sham.

**TABLE 1:** Primer sequences used.

Gene name	F primer	R primer
*Has1*	CATGGGCTATGCTACCAAGT	TCAACCAACGAAGGAAGGA
*Has2*	AGTCATGTACACAGCCTTGA	GGCAGGGTCAAGCATAGTA
*Has3*	CAGTGGACTACATCCAGAGGTG	ACTCGAAGCATCTCAATGGT
*Hyal1*	AAAGTTTGGAGAATGAAGCC	GAGAGTAGAGATGCGAAGC
*Hyal2*	TCTTCACGCGTCCCACATA	GCACTCTCACCGATGGTAGA
*Rps20*	TCTGAAGGCAAGATGGGTCAC	GTGGCGGTTGGAGCAGACG

### 2.5 Statistical analysis

Graphpad Prism Version 8 was used to graph and analyze data. All data are presented as individual data points on graphs and include the mean ± SD. Two tailed *t*‐test with 95% confidence level were used to determine significance (*n* = 4–5/group). Post hoc power analysis was conducted after the study completion to determine that at least 80% power of detecting any difference in each measured outcome at Type I error α = 0.05 was achieved using two‐sided, two‐sample *t*‐test for mean differences which yielded *N* = 6 as total sample size for two groups (i.e., three mice in each sham and OVX group to identify any differences). *p* < 0.05 was considered significant and specific *p* values are noted in graphs.

## 3 RESULTS

### 3.1 Murine bilateral OVX results in a hypoestrogenic environment and alters vaginal architecture

**FIGURE 1:**
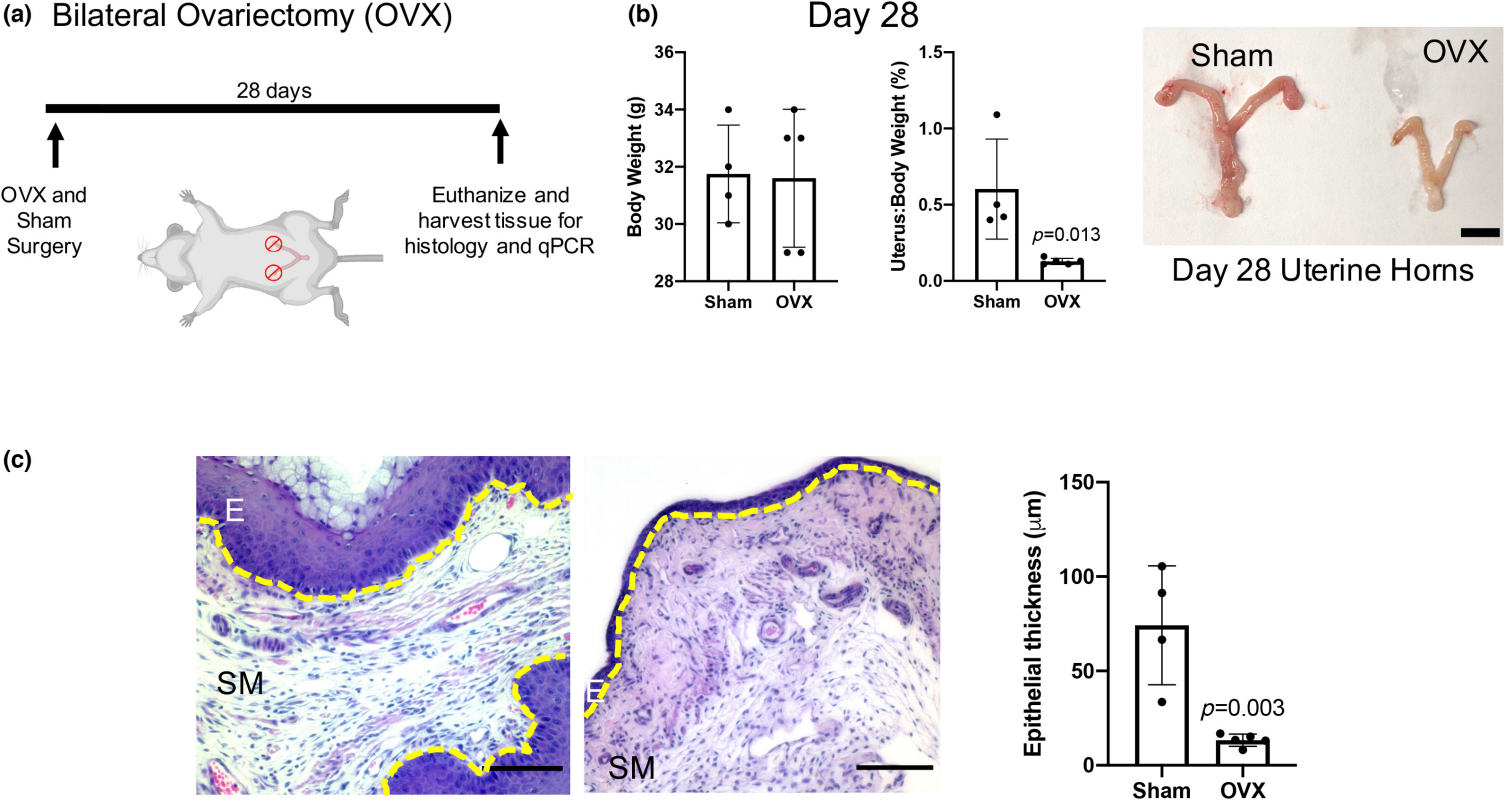
Estrogen absent milieu alters vaginal tissue. (a) The ovariectomy (OVX) schematic depicts our surgical approach and timeline between the surgical removal of the murine ovaries (or sham surgery) to the analytical end point after 28 days of recovery. Ovariectomized mice exhibit arrested circulating estrogen leading to altered phenotypes where (b) the full reproductive tract shrinks and results in a significantly reduced uterine to body weight ratio despite no change in total body weight. Scale bar, ~5 mm. (c) Moreover, the OVX vaginal epithelial layer significantly thins, observed qualitatively with H&E images and quantitatively as measured with an average of 10 lengths per sample across multiple vaginal tissue sections. Additionally, highly rugated epithelium is a hallmark characteristic of healthy vaginal tissue. However, with reduced estrogen, we observe thinned and flattened epithelium in the OVX condition. Scale bar, 100 μm.

Understanding how estrogen, produced primarily in the ovary, can affect intact vaginal tissue is vital to understanding how estrogen can affect vaginal healing. Here, murine bilateral OVX was used to model a vaginal hypoestrogenic state. Both ovaries were removed from 9‐week‐old CD1 female mice with sham‐operated mice as controls (Figure 1a). The overall body weight of OVX mice did not change compared to sham mice (Figure 1b). Due to the low basal circulating estrogen levels and difficulties in measuring them directly via ELISA (Haisenleder et al., 2011), the uterine to body weight ratio was used as a surrogate marker for decreased circulating estrogen, as has been done previously (Elliot et al., 2003). There was an 80% decrease in OVX mice compared to sham mice 1 month after surgery (Figure 1b). All the OVX vaginal tissue sections exhibit demonstrably thinner epithelium compared to respective sham tissues (Figure 1c). Sham operated mice possessed a thick, rugated epithelial layer visualized by H&E images. However, OVX vaginal tissue featured contrasting characteristics with thin and flattened epithelium presumably because of estrogen removal, which mirrors the flattened vaginal epithelium of postmenopausal women (Figure 1c). Together, by removing the ovaries from mice, and effectively depleting circulating estrogen, the entire reproductive tract and in particular the vaginal epithelial architecture was affected.

### 3.2 Estrogen signaling is altered in the OVX mice

**FIGURE 2:**
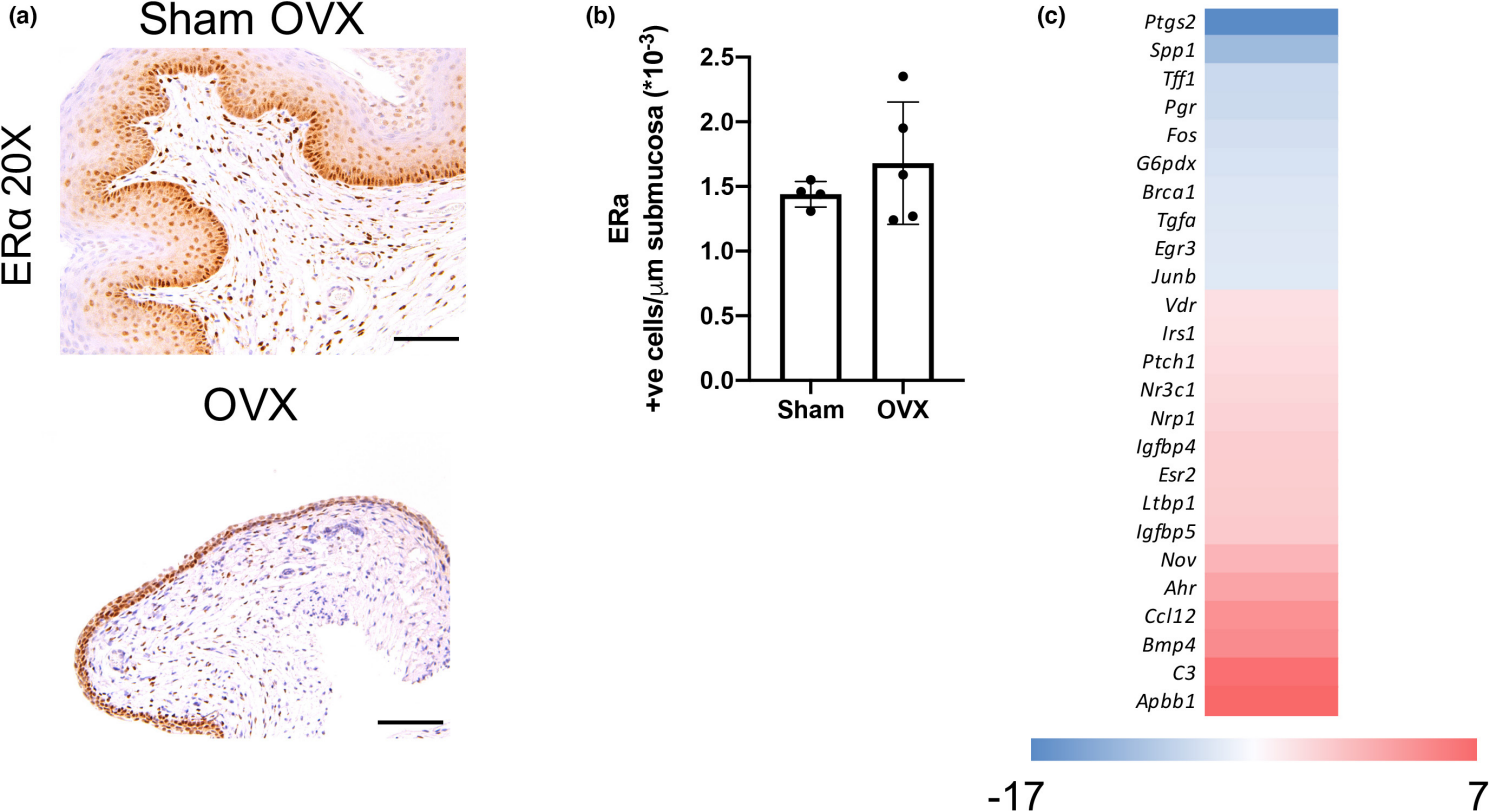
Estrogen signaling is altered in OVX murine model. (a) The immunohistochemical expression of ERα in vaginal tissue reveals differential localization across the epithelium where the highest density of ERα^+^ expression is concentrated at the basal side of the epithelium across both groups. Because the OVX condition produces only a thin epithelial layer, ERα appears ubiquitously within that thin layer of the epithelium. (b) However, the submucosa exhibits a no difference in ERα density between the OVX mice and the sham mice. (c) Overall, expression of estrogen‐related genes are altered in the OVX condition. The shifted response occurs in some upregulated genes but mostly downregulated genes in the reduced estrogen state associated with OVX. All scale bars, 100 μm.

While estrogen signals through two receptors, ERα and ERβ, ERα is the most prominently expressed in adult mouse vaginal tissue, with ERβ being undetected (Couse et al., 1997). Therefore, ERα localization was compared among OVX and sham‐operated mice. The distribution of ERα positive cells was within the basal side of the epithelium of sham mice; however, due to the thinner epithelium in the OVX mice, the full epithelium was positive for ERα staining. Additionally, the density of ERα positive cells in the submucosa was similar among sham and OVX mice (Figure 2a, b). However, differences were observed in estrogen‐related gene expression between OVX and sham‐operated mice using RNA isolated from snap frozen vaginal tissue and RT^2^ (Maslov et al., 2019) Profiler arrays for estrogen signaling. Multiple genes relating to innate immunity, including *C3* and *Ccl12* are upregulated in hypoestrogenic vaginal tissue. Estrogen is considered anti‐inflammatory, and the upregulation of pro‐inflammatory genes may be a direct result of the hypoestrogenic state (Straub, 2007). Conversely, many genes associated with estrogen signaling are suppressed under low estrogen conditions. For example, decreased *Fos* expression has been previously studied and correlated to low estrogen states in ovariectomized models as well as routinely throughout the estrus cycle when at diestrus (lowest estrogen stage). In our study, *Fos* expression is downregulated 3.4X in the OVX mice compared to sham mice (Figure 2c). Since Fos plays a role in keratinization, the results are congruent with the observed phenomena of thinner epithelium in the OVX condition.

### 3.3 HA signaling deviates from normal vaginal tissue in a low estrogen state

**FIGURE 3:**
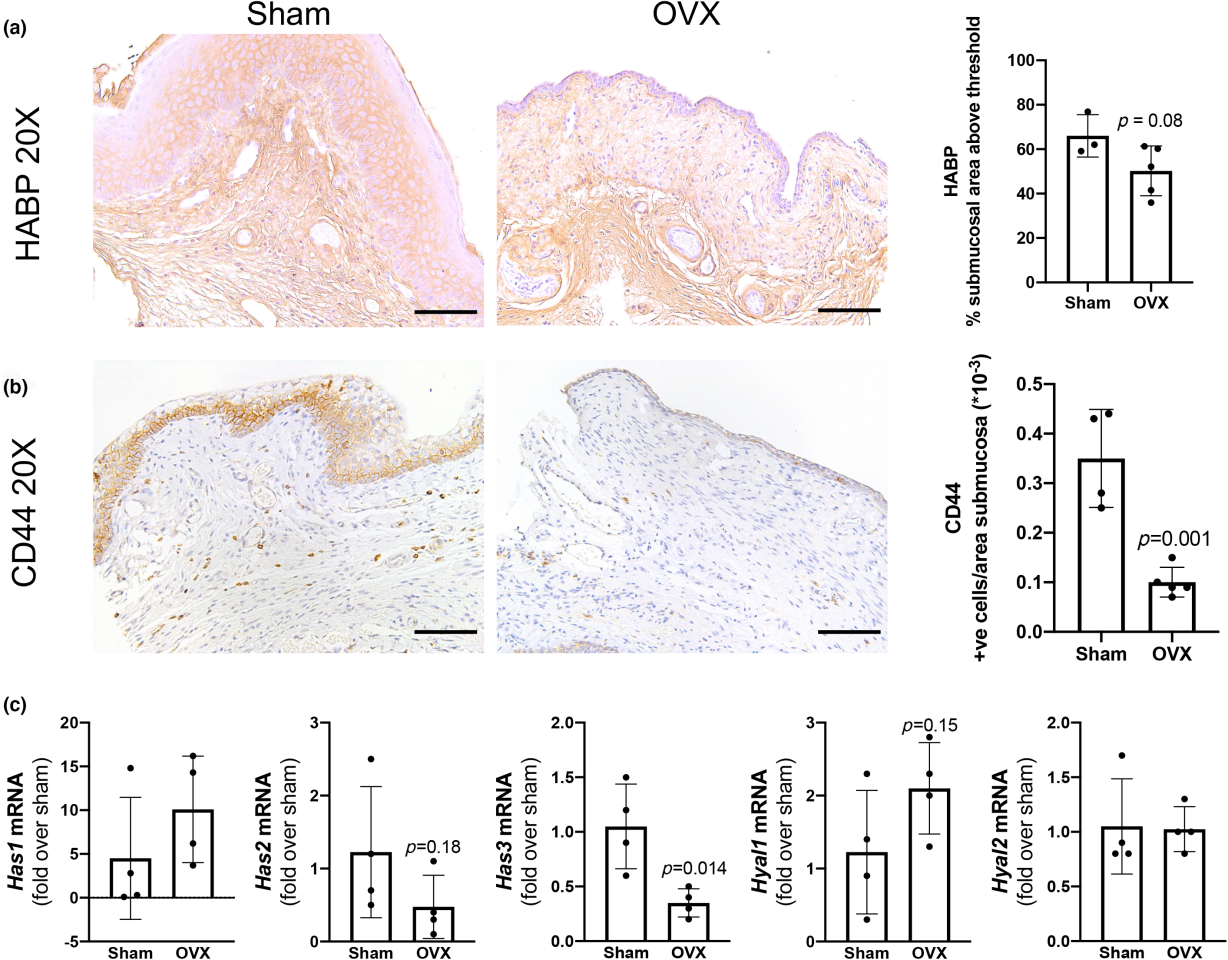
Reduction in estrogen in OVX condition disrupts hyaluronan (HA) localization and HA signaling. (a) While vaginal tissue did not exhibit significantly different hyaluronan amounts in the studied conditions, we observe altered hyaluronan localization within the submucosa. Specifically, we note decreased localization proximal to the epithelium for OVX groups. (b) CD44 expression was significantly reduced in the OVX group which implies HA signaling disruption. (c) Concomitant to the comparable hyaluronan content across groups in (a), HA synthesis and degradation gene expression results in insignificant changes but generally show a trend of elevated *Has1* (synthesis enzyme), combined with lower *Has2* and *Has3* (both synthesis enzymes), and no difference in *Hyal1* or *Hyal2* (degradation enzymes) in OVX vaginal tissue. These trends may indicate that estrogen‐reduced environments trigger dysregulated HA synthesis, degradation, and signaling. All scale bars, 100 μm.

HA is present throughout the ECM of the vagina. It is also used as a therapy for vaginal atrophy due to reduced estrogen. To investigate the effect of estrogen on vaginal HA in this murine model, HABP was used to localize tissue HA. While not significant, a trend toward a decrease in total submucosal HA in OVX mice compared to sham mice 1 month after surgery was observed (Figure 3a). Additionally, HA appears diminished in the region directly below the epithelium of the OVX tissue compared to sham mice (Figure 3a). Interestingly, while there was only a trend toward a decrease in total HA content in the submucosal regions, CD44, a primary receptor for HA, had markedly reduced expression in the low estrogen environment of the OVX group compared to sham (Figure 3b) (Misra et al., 2015). This suggested that HA signaling, via CD44, is perhaps altered in OVX. To further delineate the synthesis and degradation response of HA relative to estrogen decrease, gene expression of HA‐related genes was determined (Figure 3c). HA is synthesized by three enzymes, HAS1, HAS2, and HAS3 and has two primary degradation enzymes, Hyal1 and Hyal2. There is high variability in *Has1* expression among OVX and sham mice and no significant difference. Notably, *Has1* expression in the cervix is limited relative to the other synthases which likely extends to comparative distribution of expression in vaginal tissue and may be contributing to the high variability among mice within the same group (Akgul et al., 2014). No differences in *Has2* expression (*p* = 0.18), but a decreased *Has3* expression (*p* = 0.01) was found when comparing OVX mice to sham mice (Figure 3c). In terms of degradation, there are no significant differences in *Hyal1* or *Hyal2* expression between OVX and sham mice (Figure 3c). Taken together, the relatively small input of *Has1* combined with the decrease in *Has2* and *Has3* would suggest a decrease in HA production which could be contributing to the change in localization. Overall, these results suggest that there is altered HA localization, and the decrease in a major receptor, CD44, promotes altered HA signaling.

### 3.4 Hypoestrogenic vaginal environment alters the ECM genetic profile

**FIGURE 4:**
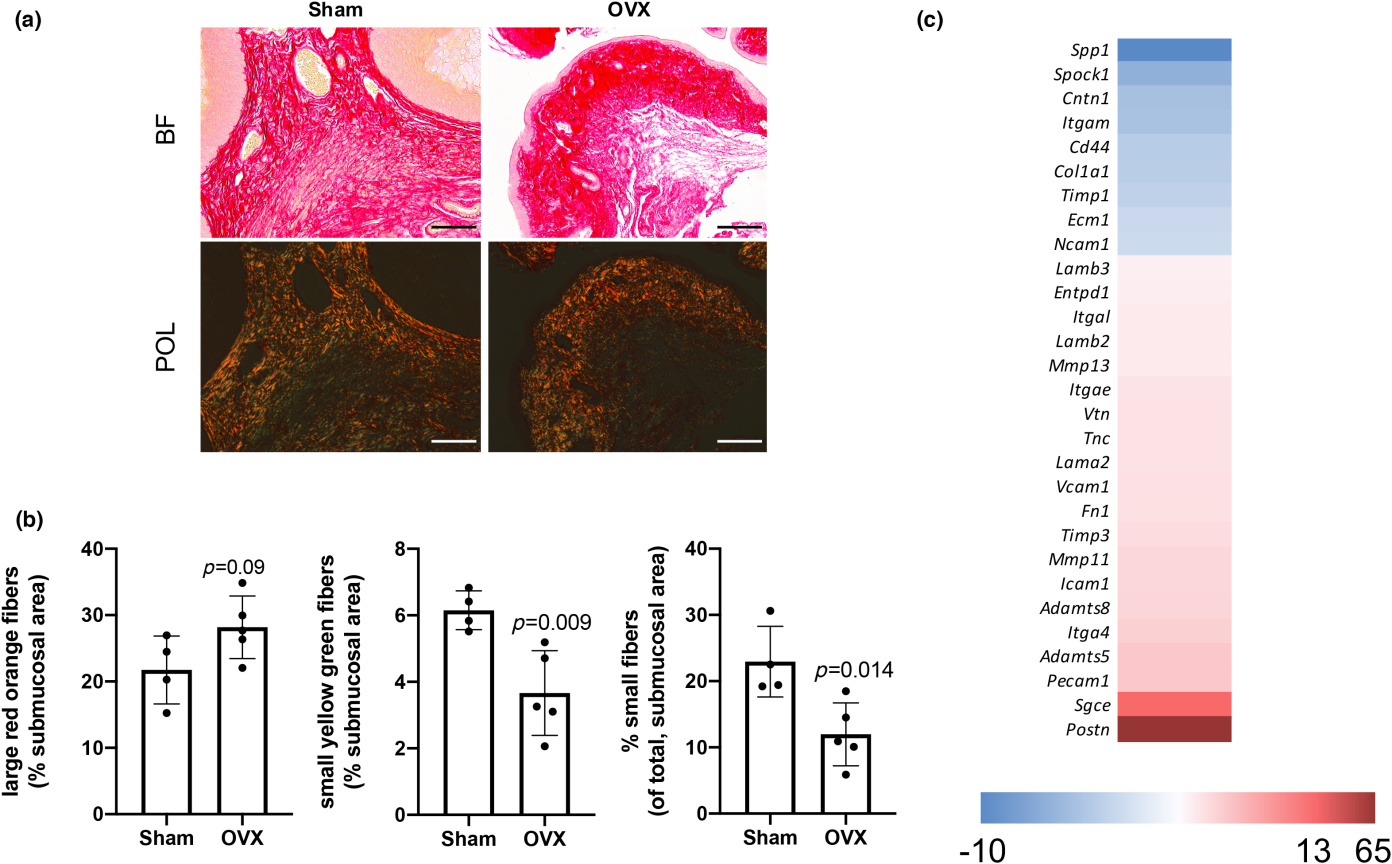
A low estrogen environment disrupts ECM and collagen profiles creating a vaginal atrophic state. (a) Picrosirius red (PSR) stained vaginal tissue was visualized using brightfield and polarized light microscopy. b) By assessing percent area covered by corresponding colorimetric parameters of red (large, Type I collagen) and yellow/green (small, Type III collagen) fibers, we showed OVX vaginal tissue sections demonstrated trends toward an increase in large fibers and a decrease in small fibers due to an estrogen‐mediated disruption of collagen turn over. We also found the percent of total collagen fibers that are considered small to be decreased in OVX mice compared to sham mice. c) Using RNA isolated from snap frozen vaginal tissue and RT^2^ (Maslov et al., 2019) Profiler inflammation arrays, we demonstrated that a low‐estrogen environment dysregulates ECM gene transcription. All scale bars, 100 μm.

Due to the interest in vaginal wound healing and the role of estrogen in vaginal ECM changes, the submucosal collagen changes and ECM gene profile in OVX mice compared to sham‐operated mice was evaluated. PSR stained vaginal tissue sections were visualized using both bright field and polarized light microscopy to differentiate between the large, red fibers most often associated with Type I collagen and the small, green/yellow fibers most often associated with Type III collagen (Figure 4a). There were trends toward more large fibers (*p* = 0.09) and significantly fewer small fibers (*p* = 0.008) in the OVX mice compared to sham mice (Figure 4b). This resulted in an alteration in the small to large fiber ratio and a decrease in the percent of total fibers that are considered small (Figure 4b). These suggests that loss of estrogen may lead to an alteration in the collagen turn over.

Additional changes in tissue remodeling gene expression in OVX versus sham‐operated mice were also found using the ECM RT^2^ (Maslov et al., 2019) Profiler arrays (Figure 4c). One of the most upregulated genes (65x) in OVX tissue is the gene for the protein, periostin (*Postn*), which plays a role in the regulation of the ECM. Generally, many of the downregulated ECM‐related genes in OVX correlate to the phenomena of vaginal tissue exhibiting overall reduced tissue strength after loss of circulating estrogen (Alvisi et al., 2019).

Taken together, these data support a vaginal tissue environment that is both altered in collagen turn over and overall tissue remodeling activity in low‐estrogen states contributing to vaginal atrophy; however, deeper studies must be completed to evaluate this in more detail as well as the contribution of the upregulation of periostin after estrogen loss.

### 3.5 Reduced inflammatory capacity associated with an estrogen poor environment

**FIGURE 5:**
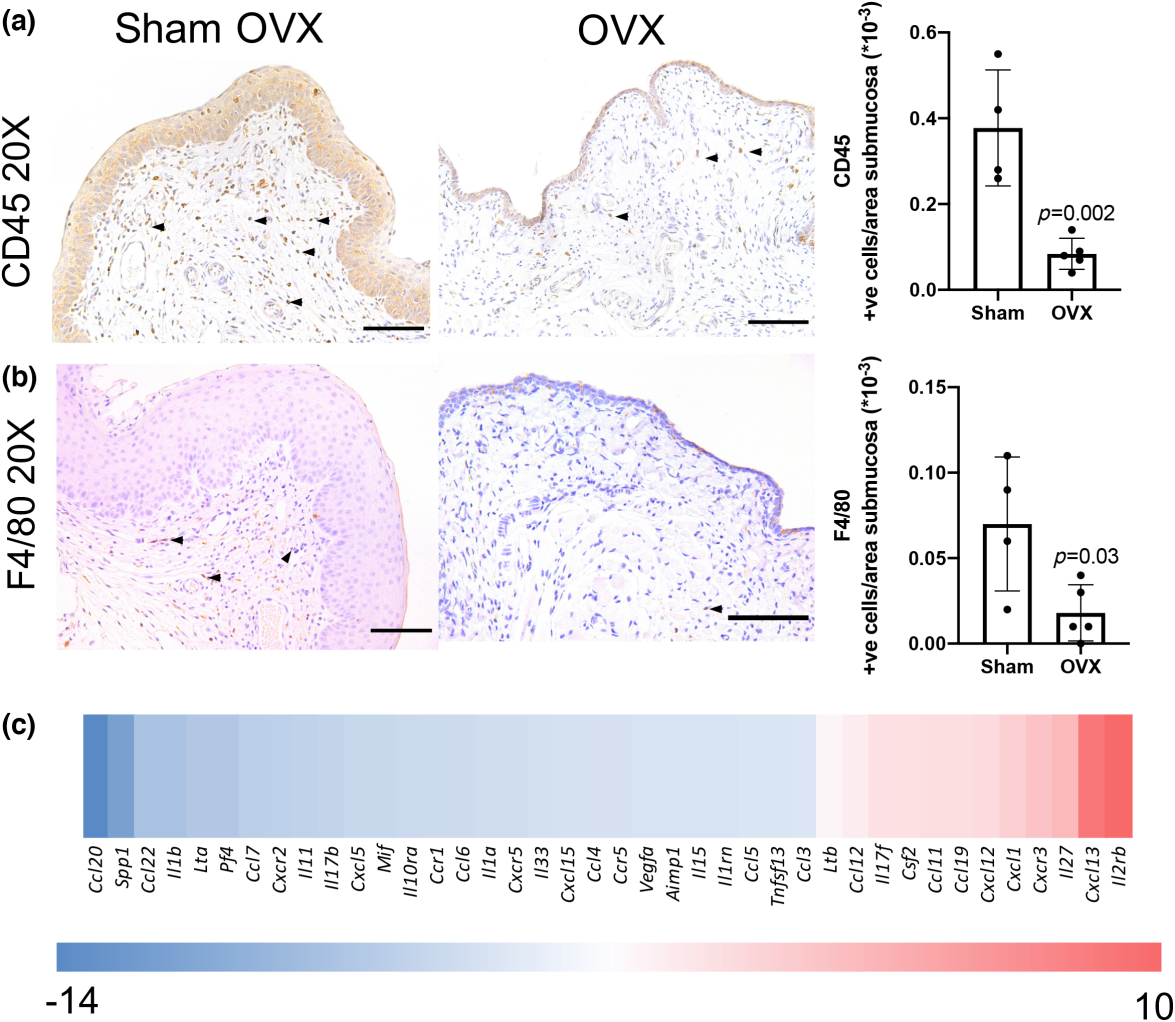
The vaginal inflammation profile is compromised following a reduction in estrogen. In vaginal tissue sections, CD45 and F4/80 positive cells were significantly reduced in OVX mice, compared to sham mice, by immunohistochemistry in (a) and (b) respectively thus potentially reducing the tissue's capacity to respond to potential insult. (c) Estrogen dependent chemotactic and inflammatory gene profiles were altered in OVX versus sham‐operated mice. All scale bars, 100 μm.

Resident and recruited inflammatory cells play a role in the regulation and alterations that occur within the ECM, not only after injury, but within healthy tissue. Therefore, the overall cellular inflammatory burden and macrophage populations was determined using IHC. Staining for CD45 to detect total leukocyte populations, and F4/80 to detect macrophages was performed. Following OVX, the number of CD45^+^ cells was significantly decreased compared to sham‐operated mice (Figure 5a). Similarly, F4/80^+^ cells were also significantly decreased in OVX mice compared to sham mice (Figure 5b). Additionally, there is a difference in estrogen dependent chemotactic and anti‐inflammatory gene targets in OVX mice compared to sham mice (Figure 5c). Multiple genes were affected, both upregulated and downregulated. *Ccl20*, a cytokine that has been shown to be a major secretion from vaginal epithelial cells and recruiter of Langerhans cells, was downregulated almost 15‐fold potentially due to the drastic reduction of the epithelial cells (Cremel et al., 2005). Additionally, the expression of pro‐inflammatory cytokines *Il1a* and *Il1b* was decreased, again likely due to decreased number of cells that synthesize it including macrophages and epithelial cells. On the contrary, there are a number of chemokines that are upregulated, including *Cxcl13* and *Cxc1*.

In sum, these data support the concept that there is a population of resident estrogen‐dependent vaginal immune cells which contribute to vaginal homeostasis. Removal of ovarian tissue and subsequent reduction in estrogen alters the basal immune defense mechanisms and may predispose the hypoestrogenic vagina to infection as noted in previously established works (Menzies et al., 2020; Qi et al., 2020).

## 4 DISCUSSION

It has been well established that loss of estrogen can alter organ systems throughout the body and contribute to diseases such as osteoarthritis and heart disease (Maslov et al., 2019; Roman‐Blas et al., 2009; Straub, 2007), but studies evaluating vaginal changes in response to decreased estrogen are limited (Han et al., 2016; Sajic et al., 2005). Here, a murine bilateral OVX model showed altered vaginal tissue, not only at the epithelial level, as observed previously (Ceccarelli et al., 2014), but also within the submucosa, specifically changes in HA and collagen turn over and CD4+ cell localization. Ovarian produced hormone withdrawal was validated in this model by utilizing the uterine to body weight ratio which serves as a surrogate for measuring estrogen reduction (Elliot et al., 2003). Women with intact estrogen signaling have a thick, rugated vaginal epithelium, however it becomes thin and friable once they undergo estrogen loss, a similar phenomenon was observed here (Ekin et al., 2011). Overall, murine ovarian loss embodies physiological characteristics seen in human vaginal tissue. Unique estrogen signaling, ECM, and inflammatory gene profiles were also found in the vaginal tissue following ovarian removal. This model will bolster mechanistic studies that will allow better understanding of the connection between estrogen, the immune system, and local vaginal environment to move toward the generation of targeted therapeutics to reduce the genitourinary and atrophic sequelae of this physiological process that virtually all women will experience.

ERα and ERβ vary with systemic estrogen level and the location, sensitivity, density, and downstream genetic targets affected by reductions in this circulating hormone, specifically in vaginal tissue, are unknown (Buchanan et al., 1998; Misra et al., 2015; O'Dwyer & Moore, 2017; Press et al., 1986). While ERβ is present and expression is altered in postmenopausal women, ERα is primarily responsible for estrogen signaling in mouse vagina (Chen et al., 1999; Dupont et al., 2000). Therefore, ERα was the focus in these studies. ERα positive cells showed consistent distribution within both the epithelium and submucosal region regardless of estrogen status. Signaling arrays show an increase in genes associated with innate immunity such as *C3* and *Ccl112* combined with downregulation of genes such as *Fos* associated with keratinization. Future studies will evaluate the connection of not only ERα density and distribution over prolonged estrogen‐absent periods but how downstream target signaling may also affect vaginal mucosa responses related to inflammation, angiogenesis, and collagen deposition. In sum, our work surmises that ERα signaling is mechanistically influenced by hormone status, which warrants further exploration; however, future studies must consider the potential role of the differential expression of ERα and ERβ between mice and human.

The intertwined nature of estrogen and HA can be understood via the dynamics of our murine model. HA is a key component of the ECM and is present in vaginal tissue (Laurent et al., 1995). While women have relied on HA gel for relief of vaginal atrophy symptoms for years, the mechanism underlying this symptomatic relief has not been fully elucidated. Potential mechanisms include retention of water to aid in lubrication, anti‐inflammatory properties, and increased tissue elasticity (Salwowska et al., 2016). Here OVX causes a nonsignificant decrease in total HA, while CD44, HA's major signaling receptor, was significantly decreased. Qualitatively, OVX mice exhibit altered localization of HA within the submucosa suggesting that the reduced estrogen state indeed disrupts the homeostasis of vaginal tissue, potentially due to dysregulated synthesis via *Has2* and *Has3*. As previously established, HAS2 is known to be a major synthesizer of high‐molecular weight HA (associated with regenerative phenotypes) (Cyphert et al., 2015). Cervical HA is synthesized primarily by HAS2, with *Has1* and *Has3* double knockout mice having comparable cervical HA as wild‐type mice (Akgul et al., 2014). Overall, our work demonstrates that when hormone cycles are disrupted, this engenders alterations in HA homeostasis and signaling via CD44.

Similar trends of estrogen's impact on HA are found in other tissue types. Skin, which is also abundant in HA, has been shown to benefit from supplemented estrogen to improve effects of accelerated skin aging after menopause (Verdier‐Sevrain et al., 2006). Within the skin, the epidermis and underlying fibroblasts work in tandem to mediate HA regulation. While the mechanisms of this crosstalk and HA‐estrogen axis are currently under investigation in various tissue types, the skin exhibits slightly reduced HAS3 in an estrogen‐reduced state similar to the observations in this study (Figure 5) (Rock et al., 2012). Moreover, estrogen was shown to increase HA content by means of paracrine signaling of the epidermal layer secreting epidermal growth factor to the underlying fibroblasts in order to induce the expression of HA synthesis enzymes (Rock et al., 2012). Vaginal tissue, seen here, is similarly affected by estrogen via its depletion to impart a reduced HA environment. Additional work to better establish the contributing role of each HAS, as well as the downstream signaling effects caused by reduced vaginal CD44 will also be considered. Lastly, the limitations of the study include focusing on CD44, while it is a major HA receptor, other receptors may be contributing to alterations in the ECM environment. In fact, activation of TLR2 and TLR4 by HA has been shown to induce antimicrobial peptides and help protect vaginal tissue from infection (Dusio et al., 2011).

Vaginal tissue ECM is composed of glycosaminoglycan (GAG, i.e., HA) and non‐GAG‐related components such as collagens I and III and elastin (Abramowitch et al., 2009). Loss of estrogen causes alterations in vaginal tissue collagen and influences tissue stiffness, alters intrinsic mechanobiological properties which in turn influence wound repair (Mao et al., 2019; Ripperda et al., 2017). In this work, alterations in collagen turn over are signaled by a decrease in the percent of the total fibers being considered small in the hypoestrogenic vaginal environment. Concomitantly, global alterations in ECM‐related genes occurred in the OVX vaginal tissue. Most significantly, periostin was upregulated almost 65X within OVX vaginal tissue compared to sham tissue. Consistently, in lung tissue, periostin is highly expressed in patients with pulmonary fibrosis (O'Dwyer & Moore, 2017). While a different tissue, it is interesting that these two mucosal tissue types share upregulated expression of periostin and may suggest the common contribution to aberrant collagen dysregulation in the respective tissue types. Even in skin, cardiac and kidney injury models, interactions between ECM glycosaminoglycan HA and matricellular protein periostin signaling have been shown to drive inflammatory and fibrogenic responses to injury by influencing fibroblast to myofibroblast differentiation and T cell polarization (Kanaoka et al., 2018; Nunomura et al., 2018; Raman et al., 2018). Interestingly, a reciprocal HA/POSTN interplay has been observed by the capacity of the latter to upregulate HAS2 expression early in the wound healing cascade, and the influence of the former to attenuate POSTN activity that has been shown in heart studies (Ghatak et al., 2014). This suggests a potential feedback mechanism between the two ECM mediators that regulates inflammatory and fibrogenesis outcomes, and has yet to be fully elucidated in vaginal remodeling. Taken together, OVX can be seen to impact ECM remodeling resulting in tissue alterations.

While postmenopausal vaginal tissue is known to have a disruption in collagen content, different models have shown conflicting results. Montoya, et al. reported OVX rats have a decrease in *Col1a1* expression compared to OVX mice treated with either systemic or vaginal estrogen (Montoya et al., 2015). Conversely Mao et al. reported increased large fiber collagen content following OVX in rats (Mao et al., 2019). Here, *Col1a1* expression (via array) was decreased and altered large to small collagen fiber ratio was noted. Perhaps this increased tendency for large, Type I collagen in the OVX condition can contribute to the increased tissue stiffness often associated with menopausal vaginal tissue (Alvisi et al., 2019). The difference may come in comparing OVX and supplemental estrogen versus comparing sham and OVX conditions contributing to the conflicting results. Another particular gene of interest, *Entpd1*, was found to be just over twofold upregulated in OVX. While little literature exists relating this gene to vaginal tissue, previous work connects this gene to lubrication disorders (Zhang et al., 2021). Further work will continue to evaluate these subtle differences as well as elucidate the relationship between estrogen and intrinsic mechanobiological properties of vaginal tissue such as tissue viscoelasticity.

The vagina is a unique organ in terms of immune tolerance. It must tolerate sperm and eventually the fetus during birth while also remain poised to defend against infections (Iijima et al., 2008). In order to maintain this balance, resident immune cells, including macrophages, are present and responsive to both the external stimuli and hormone fluctuation (Iijima et al., 2008; Sajic et al., 2005). When resident immune cells or ECM is altered due to a decrease in circulating estrogen (Rock et al., 2012), the vaginal tissue is rendered doubly at risk of injury, infection, and painful sequelae. In fact, women in hypoestrogenic states face frequent vaginal infections, irritations, and are more easily injured in their genitalia by minor traumas (Muhleisen & Herbst‐Kralovetz, 2016; North American Menopause S, 2007). In this study, we noted that the total leukocyte and resident macrophage populations were suppressed in the vaginal tissue of estrogen‐depleted mice. While exposure to estrogen therapy normally acts as an anti‐inflammatory, this reduction of immune cells in response to absent estrogen can be understood within the global suppression of vaginal homeostatic mechanisms. Decreased circulating estrogen also disrupts collagen and HA content and turnover likely altering local host defense systems. This finding reflects previous work establishing the pathogenicity of a disrupted estrogen system demonstrating heightened sensitivity to gonococcal virulence dependent on menstrual cycle estrogen nadirs (Islam et al., 2016).

In sum, the emerging concepts from this study surmise that estrogen depletion has significant effects specifically on vaginal tissue, in not only the epithelium but also estrogen signaling, HA and collagen homeostasis, and innate immunity. There are limitations of the study including the feeding of standard chow that contains phytoestrogens; however, all animals were fed the same chow and thus had similar phytoestrogen exposure. While utilizing gene arrays alone limits conclusions that can be made, there were also limitations due to the natively restricted size of murine vaginal tissue. Additionally, estrogen is not the only hormone secreted by the ovary, and conversely other tissues within the body secrete estrogen and other steroid hormones that may affect vaginal tissue. Therefore, additional studies must be completed to tease apart estrogen's role from other hormones as well as understand potential estrogen contributions from other tissues.

Overall, this work has contributed to the mechanistic underpinnings behind women's genitourinary symptoms for estrogen reduced states and will allow us and others to further leverage our discoveries on the bench with bedside applications that improve women's standard of clinical care and quality of life. Additionally, this model will be useful to help further study the connection between estrogen, innate immunity (specifically CD4+ cells), and HA and collagen turn over in other vaginal injury models such as surgical injury, radiation therapy, and fibrosis.

## 5 CONCLUSION

Developing new treatments for hypoestrogenic vaginal symptoms rely on better understanding the altered cellular and tissue environment. The bilateral OVX mouse model recapitulates the thinner epithelium, altered HA localization and signaling, and dysregulated collagen content observed in human postmenopausal vaginal tissue as well as vaginal tissue impacted from premature ovarian failure. Further, ovarian removal results in decreased basal vaginal inflammation, and we propose this could lead to lower defense mechanisms that often make postmenopausal women victims of vaginal infections. Further studies using this model will evaluate the impacts of prolonged estrogen depletion along with investigations into uncovering other mechanisms of ECM dysregulation via biomechanical perturbations. Additionally, this model can be used in other aspects of gynecologic research to establish connections between hormone signaling and wound healing, infection, and/or fibrosis. In summary, the characterization into the submucosal changes of this mouse model, specifically CD4+ cells, HA, and collagen turnover, will pave way for advancement in women's vaginal health discoveries, innovations, and treatment paradigms.

## AUTHOR CONTRIBUTIONS

Jennifer M McCracken: Conceptualization, Methodology, Validation, Formal Analysis, Investigation, Data Curation, Writing–Original Draft, Writing–Review and Editing, Visualization, Project Administration. Gisele A Calderon: Conceptualization, Methodology, Validation, Formal Analysis, Investigation, Data Curation, Writing–Original Draft, Writing–Review and Editing, Visualization, Project Administration. Quynh N Le: Data Analysis, Writing–Review and Editing. Natasha M Faruqui: Data Curation and Analysis. Swathi Balaji: Data assessment, critical review and editing of the manuscript. Julie CE Hakim: Conceptualization, Supervision, Project Administration, Funding Acquisition.

## FUNDING INFORMATION

This work was graciously supported by NIGMS 1K08GM135638–01.

## CONFLICT OF INTEREST STATEMENT

The authors have no conflict of interest to declare.

## References

[phy215762-bib-0001] Abramowitch, S. D. , Feola, A. , Jallah, Z. , & Moalli, P. A. (2009). Tissue mechanics, animal models, and pelvic organ prolapse: A review. European Journal of Obstetrics, Gynecology, and Reproductive Biology, 144(Suppl 1), S146–S158. 19285776 10.1016/j.ejogrb.2009.02.022

[phy215762-bib-0002] Akgul, Y. , Word, R. A. , Ensign, L. M. , Yamaguchi, Y. , Lydon, J. , Hanes, J. , & Mahendroo, M. (2014). Hyaluronan in cervical epithelia protects against infection‐mediated preterm birth. The Journal of Clinical Investigation, 124(12), 5481–5489. 25384213 10.1172/JCI78765 PMC4348952

[phy215762-bib-0003] Alvisi, S. , Gava, G. , Orsili, I. , Giacomelli, G. , Baldassarre, M. , Seracchioli, R. , & Meriggiola, M. C. (2019). Vaginal health in menopausal women. Medicina (Kaunas, Lithuania), 55(10), 615. 31547180 10.3390/medicina55100615 PMC6843679

[phy215762-bib-0004] Ashton‐Miller, J. A. , & Delancey, J. O. (2009). On the biomechanics of vaginal birth and common sequelae. Annual Review of Biomedical Engineering, 11, 163–176. 10.1146/annurev-bioeng-061008-124823 PMC2897058 19591614

[phy215762-bib-0005] Assaf, A. R. , Bushmakin, A. G. , Joyce, N. , Louie, M. J. , Flores, M. , & Moffatt, M. (2017). The relative burden of menopausal and postmenopausal symptoms versus other major conditions: A retrospective analysis of the medical expenditure panel survey data. American Health & Drug Benefits, 10(6), 311–321. 28975014 PMC5620512

[phy215762-bib-0006] Bachmann, G. A. , & Nevadunsky, N. S. (2000). Diagnosis and treatment of atrophic vaginitis. American Family Physician, 61(10), 3090–3096. 10839558

[phy215762-bib-0007] Buchanan, D. L. , Kurita, T. , Taylor, J. A. , Lubahn, D. B. , Cunha, G. R. , & Cooke, P. S. (1998). Role of stromal and epithelial estrogen receptors in vaginal epithelial proliferation, stratification, and cornification. Endocrinology, 139(10), 4345–4352. 9751518 10.1210/endo.139.10.6241

[phy215762-bib-0008] Cardozo, L. , Bachmann, G. , McClish, D. , Fonda, D. , & Birgerson, L. (1998). Meta‐analysis of estrogen therapy in the management of urogenital atrophy in postmenopausal women: Second report of the hormones and urogenital therapy committee. Obstetrics and Gynecology, 92(4 Pt 2), 722–727. 9764689 10.1016/s0029-7844(98)00175-6

[phy215762-bib-0009] Ceccarelli, S. , D'Amici, S. , Vescarelli, E. , Coluccio, P. , Matricardi, P. , Gioia, C. , Benedetti Panici, P. , Romano, F. , Frati, L. , Angeloni, A. , & Marchese, C. (2014). Topical KGF treatment as a therapeutic strategy for vaginal atrophy in a model of ovariectomized mice. Journal of Cellular and Molecular Medicine, 18(9), 1895–1907. 25088572 10.1111/jcmm.12334 PMC4196664

[phy215762-bib-0010] Chen, G. D. , Oliver, R. H. , Leung, B. S. , Lin, L. Y. , & Yeh, J. (1999). Estrogen receptor alpha and beta expression in the vaginal walls and uterosacral ligaments of premenopausal and postmenopausal women. Fertility and Sterility, 71(6), 1099–1102. 10360917 10.1016/s0015-0282(99)00113-2

[phy215762-bib-0011] Couse, J. F. , Lindzey, J. , Grandien, K. , Gustafsson, J. A. , & Korach, K. S. (1997). Tissue distribution and quantitative analysis of estrogen receptor‐alpha (ERalpha) and estrogen receptor‐beta (ERbeta) messenger ribonucleic acid in the wild‐type and ERalpha‐knockout mouse. Endocrinology, 138(11), 4613–4621. 9348186 10.1210/endo.138.11.5496

[phy215762-bib-0012] Cremel, M. , Berlier, W. , Hamzeh, H. , Cognasse, F. , Lawrence, P. , Genin, C. , Bernengo, J. C. , Lambert, C. , Dieu‐Nosjean, M. C. , & Delézay, O. (2005). Characterization of CCL20 secretion by human epithelial vaginal cells: Involvement in Langerhans cell precursor attraction. Journal of Leukocyte Biology, 78(1), 158–166. 15831560 10.1189/jlb.0305147

[phy215762-bib-0013] Cyphert, J. M. , Trempus, C. S. , & Garantziotis, S. (2015). Size matters: Molecular weight specificity of hyaluronan effects in cell biology. International Journal of Cell Biology, 2015, 563818. 26448754 10.1155/2015/563818 PMC4581549

[phy215762-bib-0014] Dos Santos, C. C. M. , Uggioni, M. L. R. , Colonetti, T. , Colonetti, L. , Grande, A. J. , & Da Rosa, M. I. (2021). Hyaluronic acid in Postmenopause vaginal atrophy: A systematic review. The Journal of Sexual Medicine, 18(1), 156–166. 33293236 10.1016/j.jsxm.2020.10.016

[phy215762-bib-0015] Dupont, S. , Krust, A. , Gansmuller, A. , Dierich, A. , Chambon, P. , & Mark, M. (2000). Effect of single and compound knockouts of estrogen receptors alpha (ERalpha) and beta (ERbeta) on mouse reproductive phenotypes. Development, 127(19), 4277–4291. 10976058 10.1242/dev.127.19.4277

[phy215762-bib-0016] Dusio, G. F. , Cardani, D. , Zanobbio, L. , Mantovani, M. , Luchini, P. , Battini, L. , Galli, V. , Diana, A. , Balsari, A. , & Rumio, C. (2011). Stimulation of TLRs by LMW‐HA induces self‐defense mechanisms in vaginal epithelium. Immunology and Cell Biology, 89(5), 630–639. 21102537 10.1038/icb.2010.140

[phy215762-bib-0017] Ekin, M. , Yasar, L. , Savan, K. , et al. (2011). The comparison of hyaluronic acid vaginal tablets with estradiol vaginal tablets in the treatment of atrophic vaginitis: A randomized controlled trial. Archives of Gynecology and Obstetrics, 283(3), 539–543. 20135132 10.1007/s00404-010-1382-8

[phy215762-bib-0018] Elliot, S. J. , Karl, M. , Berho, M. , Potier, M. , Zheng, F. , Leclercq, B. , Striker, G. E. , & Striker, L. J. (2003). Estrogen deficiency accelerates progression of glomerulosclerosis in susceptible mice. The American Journal of Pathology, 162(5), 1441–1448. 12707027 10.1016/S0002-9440(10)64277-0 PMC1851210

[phy215762-bib-0019] Gandhi, J. , Chen, A. , Dagur, G. , Suh, Y. , Smith, N. , Cali, B. , & Khan, S. A. (2016). Genitourinary syndrome of menopause: An overview of clinical manifestations, pathophysiology, etiology, evaluation, and management. American Journal of Obstetrics and Gynecology, 215(6), 704–711. 27472999 10.1016/j.ajog.2016.07.045

[phy215762-bib-0020] Gass, M. L. , Cochrane, B. B. , Larson, J. C. , et al. (2011). Patterns and predictors of sexual activity among women in the hormone therapy trials of the Women's Health Initiative. Menopause, 18(11), 1160–1171. 21983008 10.1097/gme.0b013e3182227ebd

[phy215762-bib-0021] Ghatak, S. , Misra, S. , Norris, R. A. , et al. (2014). Periostin induces intracellular cross‐talk between kinases and hyaluronan in atrioventricular valvulogenesis. J. Biol. Chem, 289(12), 8545–8561. 24469446 10.1074/jbc.M113.539882 PMC3961678

[phy215762-bib-0022] Guidozzi, F. (2013). Estrogen therapy in gynecological cancer survivors. Climacteric, 16(6), 611–617. 23952524 10.3109/13697137.2013.806471

[phy215762-bib-0023] Haisenleder, D. J. , Schoenfelder, A. H. , Marcinko, E. S. , Geddis, L. M. , & Marshall, J. C. (2011). Estimation of estradiol in mouse serum samples: Evaluation of commercial estradiol immunoassays. Endocrinology, 152(11), 4443–4447. 21933867 10.1210/en.2011-1501 PMC3198998

[phy215762-bib-0024] Han, N. R. , Kim, N. R. , Kim, H. M. , & Jeong, H. J. (2016). Cysteine prevents menopausal syndromes in ovariectomized mouse. Reproductive Sciences, 23(5), 670–679. 26494699 10.1177/1933719115612133

[phy215762-bib-0025] Iijima, N. , Thompson, J. M. , & Iwasaki, A. (2008). Dendritic cells and macrophages in the genitourinary tract. Mucosal Immunology, 1(6), 451–459. 19079212 10.1038/mi.2008.57 PMC2684461

[phy215762-bib-0026] Islam, E. A. , Shaik‐Dasthagirisaheb, Y. , Kaushic, C. , Wetzler, L. M. , & Gray‐Owen, S. D. (2016). The reproductive cycle is a pathogenic determinant during gonococcal pelvic inflammatory disease in mice. Mucosal Immunology, 9(4), 1051–1064. 26693700 10.1038/mi.2015.122 PMC4915993

[phy215762-bib-0027] Kagan, R. , Kellogg‐Spadt, S. , & Parish, S. J. (2019). Practical treatment considerations in the Management of Genitourinary Syndrome of menopause. Drugs & Aging, 36(10), 897–908. 31452067 10.1007/s40266-019-00700-w PMC6764929

[phy215762-bib-0028] Kanaoka, M. , Yamaguchi, Y. , Komitsu, N. , Feghali‐Bostwick, C. A. , Ogawa, M. , Arima, K. , Izuhara, K. , & Aihara, M. (2018). Pro‐fibrotic phenotype of human skin fibroblasts induced by periostin via modulating TGF‐beta signaling. Journal of Dermatological Science, 90(2), 199–208. 29433908 10.1016/j.jdermsci.2018.02.001

[phy215762-bib-0029] Laurent, C. , Hellstrom, S. , Engstrom‐Laurent, A. , Wells, A. F. , & Bergh, A. (1995). Localization and quantity of hyaluronan in urogenital organs of male and female rats. Cell and Tissue Research, 279(2), 241–248. 7534651 10.1007/BF00318480

[phy215762-bib-0030] Mao, M. , Li, Y. , Zhang, Y. , Kang, J. , & Zhu, L. (2019). Tissue composition and biomechanical property changes in the Vaginal Wall of ovariectomized young rats. BioMed Research International, 2019, 8921284. 31467917 10.1155/2019/8921284 PMC6699277

[phy215762-bib-0031] Maslov, P. Z. , Kim, J. K. , Argulian, E. , Ahmadi, A. , Narula, N. , Singh, M. , Bax, J. , & Narula, J. (2019). Is cardiac diastolic dysfunction a part of post‐menopausal syndrome? JACC Heart Failure, 7(3), 192–203. 30819374 10.1016/j.jchf.2018.12.018

[phy215762-bib-0032] McCracken, J. M. , Balaji, S. , Keswani, S. G. , & Hakim, J. C. (2021). An Avant‐Garde model of injury‐induced regenerative vaginal wound healing. Advances in Wound Care, 10(4), 165–173. 32602816 10.1089/wound.2020.1198 PMC7906868

[phy215762-bib-0033] Menzies, F. M. , Oldham, R. S. , Waddell, C. , Nelson, S. M. , & Nibbs, R. J. B. (2020). A comprehensive profile of chemokine gene expression in the tissues of the female reproductive tract in mice. Immunological Investigations, 49(3), 264–286. 31429329 10.1080/08820139.2019.1655573

[phy215762-bib-0034] Misra, S. , Hascall, V. C. , Markwald, R. R. , & Ghatak, S. (2015). Interactions between hyaluronan and its receptors (CD44, RHAMM) regulate the activities of inflammation and cancer. Frontiers in Immunology, 6, 201. 25999946 10.3389/fimmu.2015.00201 PMC4422082

[phy215762-bib-0035] Montoya, T. I. , Maldonado, P. A. , Acevedo, J. F. , & Word, R. A. (2015). Effect of vaginal or systemic estrogen on dynamics of collagen assembly in the rat vaginal wall. Biology of Reproduction, 92(2), 43. 25537371 10.1095/biolreprod.114.118638 PMC4326728

[phy215762-bib-0036] Muhleisen, A. L. , & Herbst‐Kralovetz, M. M. (2016). Menopause and the vaginal microbiome. Maturitas, 91, 42–50. 27451320 10.1016/j.maturitas.2016.05.015

[phy215762-bib-0037] Naumova, I. , & Castelo‐Branco, C. (2018). Current treatment options for postmenopausal vaginal atrophy. International Journal of Women's Health, 10, 387–395. 10.2147/IJWH.S158913 PMC6074805 30104904

[phy215762-bib-0038] Nippita, T. A. , & Baber, R. J. (2007). Premature ovarian failure: A review. Climacteric, 10(1), 11–22. 10.1080/13697130601135672 17364600

[phy215762-bib-0039] North American Menopause S . (2007). The role of local vaginal estrogen for treatment of vaginal atrophy in postmenopausal women: 2007 position statement of the North American Menopause Society. Menopause, 14(3 Pt 1), 355–369 quiz 370–351. 17438512 10.1097/gme.0b013e31805170eb

[phy215762-bib-0040] Nunomura, S. , Nanri, Y. , Ogawa, M. , Arima, K. , Mitamura, Y. , Yoshihara, T. , Hasuwa, H. , Conway, S. J. , & Izuhara, K. (2018). Constitutive overexpression of periostin delays wound healing in mouse skin. Wound Repair and Regeneration, 26(1), 6–15. 29418037 10.1111/wrr.12616 PMC5906136

[phy215762-bib-0041] O'Dwyer, D. N. , & Moore, B. B. (2017). The role of periostin in lung fibrosis and airway remodeling. Cellular and Molecular Life Sciences, 74(23), 4305–4314. 28918442 10.1007/s00018-017-2649-z PMC5659879

[phy215762-bib-0042] Palacios, S. (2009). Managing urogenital atrophy. Maturitas, 63(4), 315–318. 19493638 10.1016/j.maturitas.2009.04.009

[phy215762-bib-0043] Parish, S. J. , Nappi, R. E. , Krychman, M. L. , Kellogg‐Spadt, S. , Simon, J. A. , Goldstein, J. A. , & Kingsberg, S. A. (2013). Impact of vulvovaginal health on postmenopausal women: A review of surveys on symptoms of vulvovaginal atrophy. International Journal of Women's Health, 5, 437–447. 10.2147/IJWH.S44579 PMC3735281 23935388

[phy215762-bib-0044] Peacock, K. , & Ketvertis, K. (2022). Menopause. StatPearls Publishing. 29939603

[phy215762-bib-0045] Press, M. F. , Nousek‐Goebl, N. A. , Bur, M. , & Greene, G. L. (1986). Estrogen receptor localization in the female genital tract. The American Journal of Pathology, 123(2), 280–292. 2939725 PMC1888320

[phy215762-bib-0046] Qi, W. , Li, H. , Wang, C. , Li, H. , Fan, A. , Han, C. , & Xue, F. (2020). The effect of pathophysiological changes in the vaginal milieu on the signs and symptoms of genitourinary syndrome of menopause (GSM). Menopause, 28(1), 102–108. 32810079 10.1097/GME.0000000000001644

[phy215762-bib-0047] Raman, A. , Parnell, S. C. , Zhang, Y. , Reif, G. A. , Dai, Y. , Khanna, A. , Daniel, E. , White, C. , Vivian, J. L. , & Wallace, D. P. (2018). Periostin overexpression in collecting ducts accelerates renal cyst growth and fibrosis in polycystic kidney disease. American Journal of Physiology. Renal Physiology, 315(6), F1695–F1707. 30332313 10.1152/ajprenal.00246.2018 PMC6336984

[phy215762-bib-0048] Ripperda, C. M. , Maldonado, P. A. , Acevedo, J. F. , Keller, P. W. , Akgul, Y. , Shelton, J. M. , & Word, R. A. (2017). Vaginal estrogen: A dual‐edged sword in postoperative healing of the vaginal wall. Menopause, 24(7), 838–849. 28169915 10.1097/GME.0000000000000840 PMC5484719

[phy215762-bib-0049] Rock, K. , Meusch, M. , Fuchs, N. , et al. (2012). Estradiol protects dermal hyaluronan/versican matrix during photoaging by release of epidermal growth factor from keratinocytes. The Journal of Biological Chemistry, 287(24), 20056–20069. 22493503 10.1074/jbc.M112.353151 PMC3370189

[phy215762-bib-0050] Roman‐Blas, J. A. , Castaneda, S. , Largo, R. , & Herrero‐Beaumont, G. (2009). Osteoarthritis associated with estrogen deficiency. Arthritis Research & Therapy, 11(5), 241. 19804619 10.1186/ar2791 PMC2787275

[phy215762-bib-0051] Sajic, D. , Patrick, A. J. , & Rosenthal, K. L. (2005). Mucosal delivery of CpG oligodeoxynucleotides expands functional dendritic cells and macrophages in the vagina. Immunology, 114(2), 213–224. 15667566 10.1111/j.1365-2567.2004.02081.x PMC1782077

[phy215762-bib-0052] Salwowska, N. M. , Bebenek, K. A. , Zadlo, D. A. , & Wcislo‐Dziadecka, D. L. (2016). Physiochemical properties and application of hyaluronic acid: A systematic review. Journal of Cosmetic Dermatology, 15(4), 520–526. 27324942 10.1111/jocd.12237

[phy215762-bib-0053] Schorr, M. , & Miller, K. K. (2017). The endocrine manifestations of anorexia nervosa: Mechanisms and management. Nature Reviews. Endocrinology, 13(3), 174–186. 10.1038/nrendo.2016.175 PMC5998335 27811940

[phy215762-bib-0054] Straub, R. H. (2007). The complex role of estrogens in inflammation. Endocrine Reviews, 28(5), 521–574. 17640948 10.1210/er.2007-0001

[phy215762-bib-0055] Verdier‐Sevrain, S. , Bonte, F. , & Gilchrest, B. (2006). Biology of estrogens in skin: Implications for skin aging. Experimental Dermatology, 15(2), 83–94. 16433679 10.1111/j.1600-0625.2005.00377.x

[phy215762-bib-0056] Zerbinati, N. , & Calligaro, A. (2018). Calcium hydroxylapatite treatment of human skin: Evidence of collagen turnover through picrosirius red staining and circularly polarized microscopy. Clinical, Cosmetic and Investigational Dermatology, 11, 29–35. 29391818 10.2147/CCID.S143015 PMC5772396

[phy215762-bib-0057] Zhang, J. , Zhang, J. , Cong, S. , Feng, J. , Pan, L. , Zhu, Y. , Zhang, A. , & Ma, J. (2021). Transcriptome profiling of lncRNA and co‐expression network in the vaginal epithelial tissue of women with lubrication disorders. PeerJ, 9, e12485. 34824921 10.7717/peerj.12485 PMC8590395

